# Performance evaluation on an air-cooled heat exchanger for alumina nanofluid under laminar flow

**DOI:** 10.1186/1556-276X-6-488

**Published:** 2011-08-09

**Authors:** Tun-Ping Teng, Yi-Hsuan Hung, Tun-Chien Teng, Jyun-Hong Chen

**Affiliations:** 1 Department of Industrial Education, National Taiwan Normal University, No. 162, Section 1, He-ping East Road, Da-an District, Taipei City 10610, Taiwan, Republic of China; 2 Department of Mechatronic Technology, National Taiwan Normal University, No. 162, Section 1, He-ping Eeast Road, Da-an District, Taipei City 10610, Taiwan, Republic of China

**Keywords:** alumina (Al2O3), heat exchange capacity, laminar flow, nanofluid, pressure drop

## Abstract

This study analyzes the characteristics of alumina (Al_2_O_3_)/water nanofluid to determine the feasibility of its application in an air-cooled heat exchanger for heat dissipation for PEMFC or electronic chip cooling. The experimental sample was Al_2_O_3_/water nanofluid produced by the direct synthesis method at three different concentrations (0.5, 1.0, and 1.5 wt.%). The experiments in this study measured the thermal conductivity and viscosity of nanofluid with weight fractions and sample temperatures (20-60°C), and then used the nanofluid in an actual air-cooled heat exchanger to assess its heat exchange capacity and pressure drop under laminar flow. Experimental results show that the nanofluid has a higher heat exchange capacity than water, and a higher concentration of nanoparticles provides an even better ratio of the heat exchange. The maximum enhanced ratio of heat exchange and pressure drop for all the experimental parameters in this study was about 39% and 5.6%, respectively. In addition to nanoparticle concentration, the temperature and mass flow rates of the working fluid can affect the enhanced ratio of heat exchange and pressure drop of nanofluid. The cross-section aspect ratio of tube in the heat exchanger is another important factor to be taken into consideration.

## Introduction

As technology and energy products require higher standards of function and performance, the problem of heat dissipation is becoming a significant issue in many appliances. Using a working fluid with high heat transfer performance is a topic worthy of research, as it may solve this problem without costly changes in the structure of the equipment. Many researchers have recently investigated the issue of nanofluid thermal properties. Many studies show that nanofluids can enhance heat conduction performance due to their higher thermal conductivity than base fluids [1-6]. However, heat convection characteristics must also be considered in practical heat exchange applications. Many researchers have focused on heat transfer properties of convection for a single pipe with the different structures, and investigated the parameters of nanoparticles added, pipe cross-section structure, materials and concentration of nanofluid, flow conditions, and other factors [7-14].

Palm et al. [15] reported that the water/Al_2_O_3 _nanofluid with concentration of 4 vol.% enhanced the average wall heat transfer coefficient by 25% compared to base liquid in 2006. Furthermore, the average heat transfer coefficient increased with an increase in wall heat flux due to a decrease in the wall shear stress.

Nguyen et al. [16] used Al_2_O_3 _nanofluid with different nanoparticle sizes (36 and 47 nm) in an electronic liquid cooling system. The heat convective coefficient was enhanced by a maximum of 40% at an added particle concentration of 6.8 vol.%. The heat convective coefficient of the added nanoparticle size 36 nm was higher than that of 47 nm at the same concentration. These results show that nanofluid improve the heat transfer performance for electronic liquid cooling system, and smaller nanoparticles added to the based liquid more effectively enhanced the heat convective coefficient. Chein and Chuang [17] applied CuO/water nanofluid to a microchannel heat sink (MCHS) and found that a nanoparticle concentration of 0.2 to 0.4 vol.% enhanced the cooling performance of CuO/water nanofluid. Their experimental results show that the CuO/water nanofluid had low thermal resistance at lower flow rate (10 and 15 ml/min), and higher resistance at higher flow rate (20 ml/min). These results indicate that the flow rate is a very important factor to affect the heat convective performance of a nanofluid.

Kulkarni et al. [18] studied the specific heat of Al_2_O_3_/ethylene glycol and water (EG/W) nanofluid and its effect on the cogeneration efficiency of a 45-kW diesel electric generator (DEG) in 2008. Their experimental results show that applying nanofluid reduced cogeneration efficiency due to a decrease in the specific heat of the nanofluid. Further, the efficiency of waste heat recovery in the heat exchanger increased due to the higher convective heat transfer coefficient of the nanofluid.

Pantzali et al. [19] adopted a 4 vol.% CuO nanofluid to investigate the effects of using nanofluid in a miniature plate heat exchanger with a modulated surface through both experimental and numerical calculations. Their results demonstrate that the CuO nanofluid enhanced the heat transfer rate and total heat transfer coefficient, and suggested that the required flow rate of nanofluid was lower than that of water to keep lower pressure drop. Jung et al. [20] studied the convective heat transfer coefficient and friction factor of Al_2_O_3_-water/ethylene glycol (50:50) nanofluid with different concentrations (0.6, 1.2, 1.8 vol.%) in rectangular microchannels. They also measured the Al_2_O_3 _nanoparticles size of 170 nm in nanofluid using light scattering equipment. The convective heat transfer coefficient of the Al_2_O_3 _nanofluid at 1.8 vol.% increased 32% compared to the base liquid without a major friction loss in a laminar flow regime (5 < Re < 300). The Nusselt number increased as the Reynolds number increased in a laminar flow regime. Nnanna et al. [21] adopted Al_2_O_3_/water nanofluid for heat dissipation in the heat exchanger of a thermoelectric module. The nanoparticle size and concentration of added nanoparticles in that study were 27 nm and 2 vol.%, respectively. The average thermal contact resistance was 0.18°C/W and 0.12°C/W for the deionized water and nanofluid, respectively. Duangthongsuk and Wongwises [22] reported an experimental study on forced convective heat transfer under varied heat flux boundary conditions and pressure drop characteristics of a nanofluid with 0.2 vol.% TiO_2 _nanoparticles (*d*_p _= 21 nm) flowing in a horizontal double-tube counter flow heat exchanger under turbulent flow regimes. Their results show that the convective heat transfer coefficient of nanofluid is approximately 6% to 11% higher than that of the base liquid. The heat transfer coefficient of the nanofluid increased as the mass flow rate of the water and nanofluid increased.

Abu-Nada et al. [23] used an efficient finite-volume method to study the heat transfer characteristics of natural convection for CuO/EG/water nanofluid in a differentially heated enclosure. They presented various results for the streamline and isotherm contours and the local and average Nusselt numbers for a wide range of Rayleigh numbers (Ra = 10^3 ^to approximately 10^5^), nanoparticle concentrations (0 <*ϕ *< 6 vol.%), and enclosure aspect ratios (1/2 ≦ *A *≦ 2). Their results show that the enclosure aspect ratio had significant effects on the behavior of the average Nusselt number, which decreased as the enclosure aspect ratio increased. Ho et al. [24] investigated the forced convective cooling performance of a copper MCHS with Al_2_O_3_/water nanofluid as the coolant under laminar flow conditions (Re = 226 to approximately 1,676). Their results show that the dynamic viscosity and friction factor increased due to dispersing the alumina nanoparticles in water. The MCHS with Al_2_O_3_/water nanofluid also had higher average heat transfer coefficient, lower thermal resistance, and lower wall temperature at high pumping power. Feng and Kleinstreuer [25] presented numerical simulations for heat transfer between parallel disks with an Al_2_O_3_/water nanofluid flow. Their results indicate that the nanofluid had smoother mixture flow fields and temperature distributions. The Nusselt number increased with higher nanoparticle volume fraction, smaller nanoparticle size, reduced disk spacing, and larger inlet Reynolds number under a realistic thermal load. They also proposed the correlation of critical radial distance and minimization of total entropy generation analysis. Jwo et al. [26] adopted Al_2_O_3_/water nanofluid for heat dissipation experiments in a multi-channel heat exchanger (MCHE) to simulate its application to electronic chip cooling system. Results show that the overall heat transfer coefficient ratio was higher at higher nanoparticle concentrations. In addition, when the input temperature of nanofluid flowing into MCHE was lower, the mass flow rate had a greater effect on the overall heat transfer coefficient ratio than concentration. Farajollahi et al. [27] performed an experimental analysis to study heat transfer of nanofluid in a shell and tube heat exchanger. They used nanofluid Al_2_O_3_/water and TiO_2_/water nanofluid under turbulent flow conditions to investigate the effects of the Peclet number, volume concentration of suspended particles, and particle type on heat transfer characteristics. Their results indicate that the addition of nanoparticles to the base fluid enhances heat transfer performance. Notice that heat transfer characteristics of nanofluid increased significantly with the Peclet number. TiO_2_/water and Al_2_O_3_/water nanofluid exhibited better heat transfer behavior at lower and higher volume concentrations, respectively. The experimental results above are also in agreement with the predicted values of available correlation at the lower volume fractions of the nanoparticle.

Firouzfar et al. [28] recently used a methanol/Ag nanofluid to fill a thermosyphon heat exchanger and compared its effectiveness and energy saving with that of pure methanol. Their experimental results show that methanol/Ag nanofluid achieved an energy savings of approximately 8.8-31.5% for cooling and 18-100% for reheating the supply air stream in an air conditioning system, respectively. Zamzamian et al. [29] investigated the effects of forced convective heat transfer coefficient with Al_2_O_3_/EG and CuO/EG nanofluid in double pipe and plate heat exchangers. Their results indicate that increasing the nanoparticle concentration and temperature could enhance the convective heat transfer coefficient of nanofluid, leading to a 2% to 50% enhancement in convective heat transfer coefficient of the nanofluid.

The literature review above clearly shows that using nanofluid can effectively improve the heat convective performance, but will also increase the pipeline pressure drop and pumping energy. Using nanofluid with a high heat convective performance for heat exchange can help reduce the volume of the heat exchanger. In addition, using nanofluid with higher heat transfer performance instead of the traditional working fluid for cooling can reduce the demand and cost of cooling system modifications. Most of the nanofluids used in previous studies were used in single pipe heat transfer, microchannel heat sinks, plate heat exchangers, double-tube heat exchangers, or heated enclosures, and seldom used in air-cooled heat exchangers. Since the ultimate goal of radiators is to discharge heat into the atmosphere, and the air-cooled heat exchanger is widely used in automotive, air conditioning, proton exchange membrane fuel cell (PEMFC) and electronic chip cooling, and is therefore a worthy research direction. This study uses a two-step synthesis method to make Al_2_O_3_/water nanofluid, which can be used as coolant in an air-cooled heat exchanger to heat dissipation. Identifying the differences in nanofluid weight fractions, mass flow rates, and temperature effects on heat exchange performance and pressure drop of the air-cooled heat exchanger makes it possible to evaluate the feasibility of applying Al_2_O_3_/water nanofluid to PEMFC heat dissipation or electronic chip cooling in the future.

### Calculation for heat exchange and flow conditions

This section evaluates the heat exchange capacity of the working fluid for an air-cooled heat exchanger based on the measured inlet and outlet temperature difference (*T_i_*-*T_o_*) for different mass flow rates () and specific heat (*c_p, f_*). The heat exchange capacity () of the heat exchanger can be written as follows:(1)

Under the condition of actual application, the cross-section of pipe is not circular, so modification is needed. The characteristic length of a non-circular cross-section is called hydraulic radius (*R*), and can be expressed as(2)

where *A *is the area of cross-section, and WP is the wetter perimeter (rectangle side lengths equal to *a *and *b*, then WP = 2*a *+ 2*b*).

The Reynolds number (Re) of the flow in the non-circular cross-section pipe can be expressed as(3)

According to the concept of solid-liquid mixture, the density (*ρ*_nf_) and specific heat (*c_p_*_, nf_) of the Al_2_O_3_/water nanofluid is given by Equations 4 and 5, with volume fraction (*ϕ*), bulk fluid density (*ρ*_bf_), nanoparticle density (*ρ_p_*), bulk fluid specific heat (*c_p, bf_*), and nanoparticle specific heat (*c_p, p_*) [2,30,31]:(4)(5)

The volume fraction (*ϕ*) of the Al_2_O_3_/water nanofluid is given by Equation 6, with bulk fluid weight (*W*_bf_), nanoparticle weight (*W_p_*) and nanofluid weight (*W*_nf_):(6)

Equation 6 can be used to convert the weight fraction to volume fraction to calculate the density and specific heat of nanofluid by Equations 4 and 5. The density and specific heat of nanofluid and experimental data can then be used to calculate the Reynolds number and heat exchange capacity for the nanofluid.

### Preparation of sample and experimental design

#### Preparation of alumina nanofluid

The base liquid was prepared by adding 0.2 wt.% of cationic dispersant (water-soluble chitosan) to distilled water as a dispersant to obtain good suspension for nanofluid. The Al_2_O_3_/water nanofluid produced by two-step synthesis method was then used as the experimental sample, and homogenization, electromagnetic agitation, and ultrasonic vibration were alternately used to disperse the Al_2_O_3 _nanoparticles into three weight fractions (0.5, 1.0, 1.5 wt.%) in the base liquid. The reason for using a lower concentration of nanofluid was to avoid blocking pipes and an overly high pressure drop caused by the sedimentation of nanoparticles and increased viscosity from a high concentration of nanofluid. The Al_2_O_3_/water nanofluid used in this study contains commercial nanoparticles (Al-13P, Yong-Zhen Technomaterial, Taipei, Taiwan). The real density of Al_2_O_3 _nanoparticles is approximately 3,880 kg/m^3^, which can be converted to be weight fraction and volume fraction by Equation 6.

**Figure 1:**
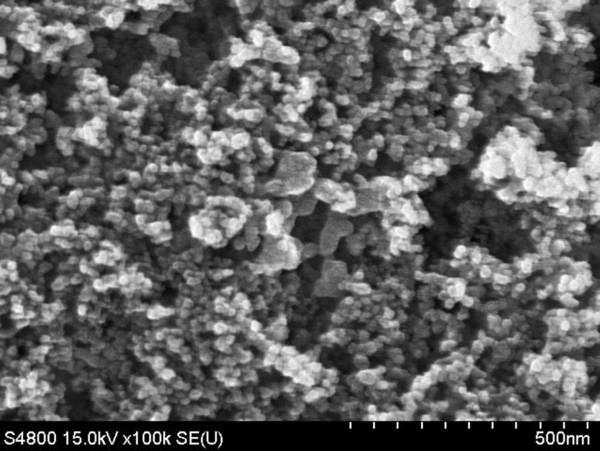
**FE-SEM images of Al_2_O_3 _nanoparticles**.

**Figure 2:**
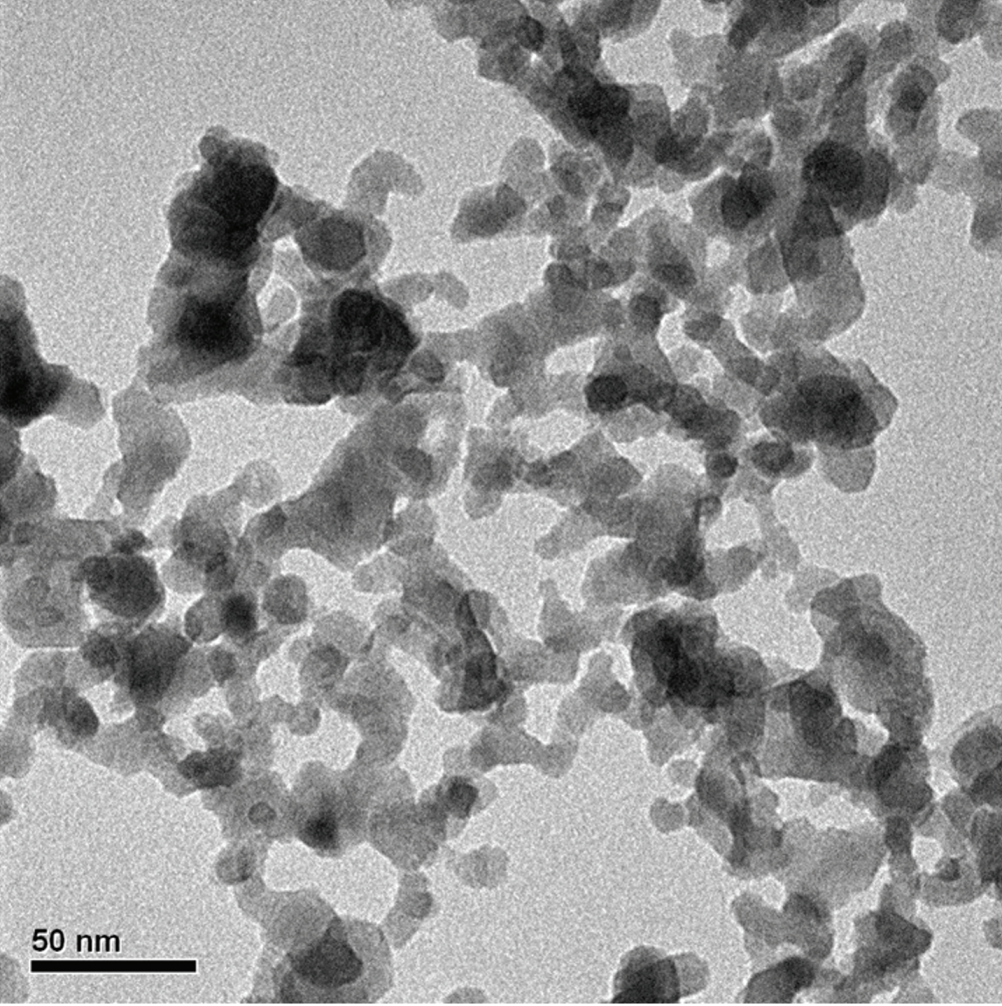
**TEM images of Al_2_O_3 _nanoparticles**.

**Figure 3:**
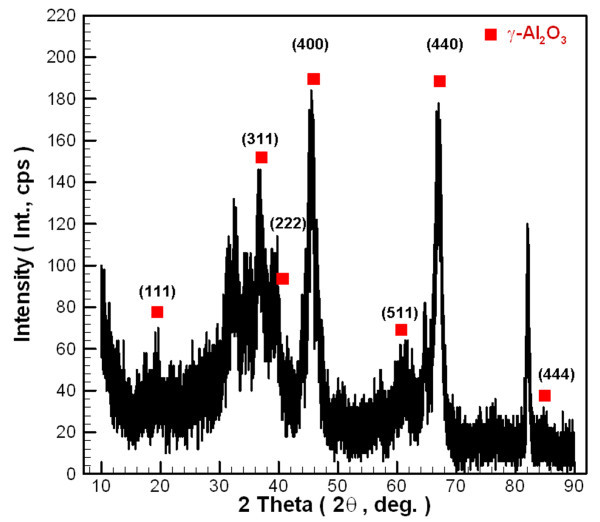
**XRD patterns of Al_2_O_3 _nanoparticles**.

Figures 1 and 2 respectively show field emission scanning electron microscope (FE-SEM, S-4800, Hitachi, Tokyo, Japan) and transmission electron microscope (TEM, H-7100, Hitachi) photographs of Al_2_O_3 _nanoparticles. These figures show that the nanoparticles exhibit an aggregate phenomenon, and the primary particle size is about 20 nm. The crystalline phase of Al_2_O_3 _nanoparticle was determined by X-ray Diffraction (XRD, APEX II, Kappa CCD, Monrovia, CA, USA). All peaks were measured by XRD and compared with those of the joint committee on powder diffraction standards data (PCPDFWIN 2.4, JCPDS-ICDD, Newtown Square, PA, USA) [32] (Figure 3). This figure confirms that the material used in this study was γ-alumina. All the completed experimental samples were allowed to remain static for 7 days to confirm suspension performance. Spectrometer analysis confirmed that the concentration of Al_2_O_3_/water nanofluid changed less than 5%.

#### Experimental procedure and design

This study investigates whether the Al_2_O_3_/water nanofluid can be used for PEMFC heat dissipation or electronic chip cooling in the future. Thus, the temperature of the test samples was set at 30°C to approximately 60°C to simulate the most common cooling temperature range in electronic cooling and PEMFC heat dissipation.

Firstly, in the thermal conductivity and viscosity experiments, a thermostatic bath (D-620, DengYng, Taipei, Taiwan) was stabilized the temperature of the sample until it reached the expected temperature (20°C to approximately 60 ± 0.5°C). A thermal properties analyzer (KD-2 Pro, Decagon Devices, Inc., Pullman, WA, USA) and rheometer (DVIII+, Brookfield, Middleboro, MA, USA) were then used to measure the thermal conductivity and viscosity in the nanofluid at various weight fractions and sample temperatures. The suspended particle size of Al_2_O_3_/water nanofluid was then measured using a dynamic light scattering (DLS) size/zeta potential analyzer (SZ-100, HORIBA, Kyoto, Japan) to determine clustering and suspension performance.

**Figure 4:**
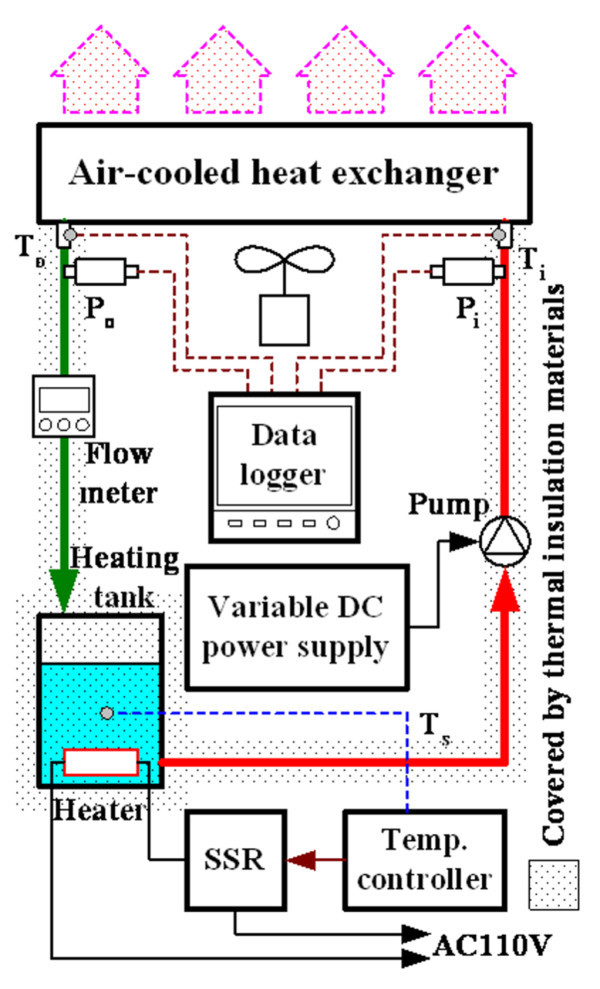
**Experimental setup for heat exchange capacity measurement**.

**Figure 5:**
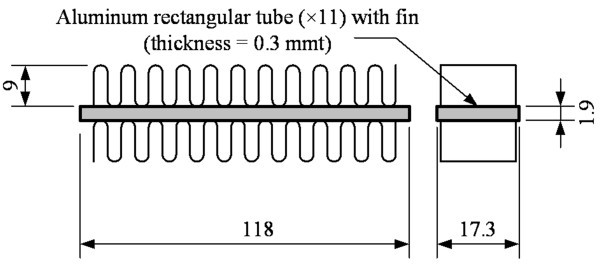
**Construction of the rectangular tube in air-cooled heat exchanger**.

The heat exchange and pressure drop experiments in this study used a heated tank to simulate the heating source, and evaluated the cooling performance of nanofluid using air-cooled heat exchangers under the conditions for different concentrations, temperatures, nanofluid mass flow rates. Figure 4 shows the experimental setup for the heat exchange capacity experiment. Figure 5 shows the construction of the rectangular tube in the air-cooled heat exchanger used in this study. After 2,200 ml of test samples were poured into a 2.5-liter acrylic tank and the sample temperature was controlled by a PID temperature controller (TTM-J4, TOHO, Japan) with SSR (SSR-40DA, Manax, Taiwan) and heater (300 W), the nanofluid was pumped to an air-cooled heat exchanger for circulation.

The heat exchange capacity of the liquid side was calculated based on measurements of the temperature difference and flow rate between the inlet and outlet of the liquid of heat exchanger. The air-cooled heat exchanger was made of aluminum, and its structure was of finned-tube type assembled with 11 rectangular tubes at 118 × 17.3 × 1.9 mm (*L *× *W *× *H*) each. The effective internal cross-sectional area was 2.17 × 10^-5 ^m^2^. The pipe was covered by thermal insulation material at the thickness of 1.5 cm to reduce the influence of heat dissipation from other components. The mass flow rate of liquid side was controlled by the input voltage (GPC-6030D, GWINSTEK, Taipei, Taiwan) of circulating pump (MCP-655, Swifttech, USA). This experiment used a temperature controller to stabilize the temperature of the sample until it reached the expected temperature (30°C, 40°C, 50°C, and 60 ± 0.5°C). An environmental control system maintained the temperature and relative humidity at 25 ± 1°C and 60 ± 5% to ensure the constant environmental conditions at the air side of heat exchanger, and kept the air side conditions of each experiment the same under fixed air flow rate. A multifunction meter (Testo-400, Testo, Lenzkirch, Germany) monitored the environmental conditions to ensure the stability of the experiment. A data logger (TRM-20, TOHO, Japan), a pressure transducer (JPT-131LJ, Jetec, Taichung, Taiwan) and a flow meter (NF05, Aichi Tokei, Nagoya, Japan) were also employed to measure the temperature, pressure, and flow rate to coordinate with the relevant equations to calculate the heat exchange capacity.

#### Data and uncertainty analysis

The results of heat exchange capacity and pressure drop obtained with the distilled water were used as baseline values (*D*_bf_) to allow easy comparison of experimental data after changing the Al_2_O_3_/water nanofluid (*D*_nf_). In other words, the experimental data obtained with the Al_2_O_3_/water nanofluid was compared with baseline values. The differences between the before-and-after changes by the Al_2_O_3_/water nanofluid were presented as proportions (ER), and can be calculated as follows:(7)

The uncertainty of the experimental results was determined based on the measurement deviation of the parameters, including thermal conductivity, viscosity, flow rate, input voltage, weight, and temperature. The thermal conductivity experiment calculated the thermal conductivity based on readings of the thermal property analyzer (*k*). The weight (*W*) of nanoparticles was measured by a precise electric balance (XT-620 M, Precisa Dietikon, Switzerland). The temperature of the isothermal bath (*T*) was measured by resistance temperature detector (RTD, pt-100).(8)

The precision of the thermal property analyzer is ± 5%. The accuracy of the precise electric balance is ± 0.01 g. The precision of the RTD is ± 0.5°C Hence, the uncertainty of the thermal conductivity experiment was calculated to be less than ± 5.6%.

The viscosity experiment calculated the viscosity based on readings of the rheometer (*μ*). The weight (*W*) of nanoparticles was measured by a precise electric balance. The temperature of isothermal bath (*T*) was measured by resistance temperature detector (RTD, pt-100).(9)

The precision of the rheometer is ± 1%. The accuracy of the precise electric balance is ± 0.01 g. The precision of the RTD is ± 0.5°C Hence, the uncertainty of the viscosity experiment was calculated to be less than ± 2.7%.

The Reynolds number (Re) experiment on nanofluid measured the flow velocity rate (*v_m_*) using a flow meter and cross-sectional area. The viscosity was determined based on readings of the rheometer (*μ*). The weight (*W*) of nanoparticles was measured by a precise electric balance, and the temperature was determined using thermocouples (*T*; T-type). Ignoring the calculation deviations generated by Equations 4, 5, and 6 and tube size, the uncertainty of these experimental results can be expressed as follows:(10)

The accuracy of the flow meter is ± 2.0%. The precision of the rheometer is ± 1%. The accuracy of the precise electric balance is ± 0.01 g. The accuracy of the thermocouple is ± 0.5°C. Therefore, the uncertainty of the Re experiment was calculated to be less than ± 3.4%.

The pressure drop experiment on nanofluid measured the mass flow rate () using a flow meter and density of liquid. The pressure drop (dP) of the liquid was measured by a pressure transducer. The weight (*W*) of nanoparticles was measured by a precise electric balance, and temperature was determined using thermocouples (T-type, *T*). Ignoring the calculation deviations generated by Equations 4, 5. and 6, the uncertainty of these experimental results can be expressed as follows:(11)

The accuracy of the flow meter is ± 2.0%. The accuracy of the pressure transducer is ± 0.5%. The accuracy of the precise electric balance is ± 0.01 g. The accuracy of the thermocouple is ± 0.5°C. Therefore, the uncertainty of the pressure drop experiment was calculated to be less than ± 3.3%.

The heat exchange capacity experiment on nanofluid measured the mass flow rates () using a flow meter and density of liquid. The weight (*W*) of nanoparticles was measured by a precise electric balance, and temperature was determined using thermocouples (T-type, *T*). Ignoring the calculation deviations generated by Equations 1, 4, 5, and 6), the uncertainty of experimental results can be expressed as follows:(12)

The accuracy of the flow meter is ± 2.0%. The accuracy of the precise electric balance is ± 0.01 g. The accuracy of the thermocouple is ± 0.5°C. Therefore, the uncertainty of the heat exchange capacity experiment was calculated to be less than ± 3.3%.

## Results and discussion

**Figure 6:**
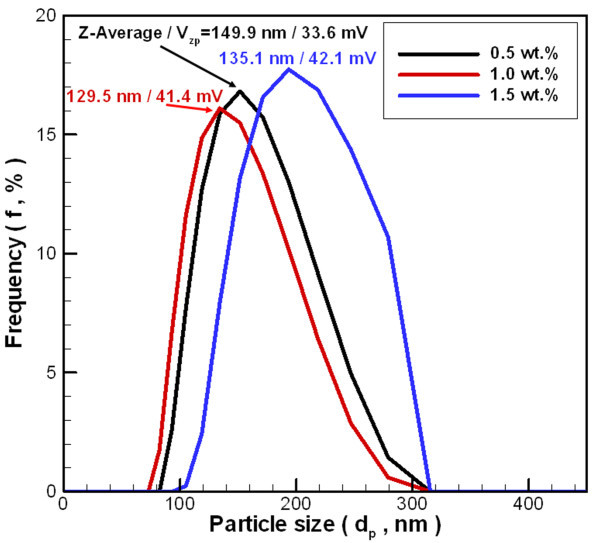
**The particle size distribution for the Al_2_O_3 _nanoparticles suspended in base liquid**.

This study uses a dynamic light scattering size/zeta potential analyzer to determine the average size of the nanoparticle suspended in base liquid. Figure 6 shows the particle size distribution of the Al_2_O_3 _nanoparticles suspended in base liquid. The z-average particle size and zeta potential is 149.9/33.6 mV, 129.5/41.4 mV, and 135.1/42.1 mV at 0.5 wt.%, 1.0 wt.%, and 1.5 wt.%, respectively. These distributions have a single peak, and the particle size distribution concentrated between 80 to approximately 310 nm. The tested particle size from DLS size/zeta potential analyzer exceeded the particle size observed by FE-SEM and TEM for the following two reasons: (1) The particle size analyzer measures the nanoparticle size based on the principle of dynamic light scattering, and is therefore affected by the viscosity and refractive index of solution. This is because viscosity and refractive index both affect the mobility of nanoparticles in solution, causing deviations in the measurement. (2) Because the agglomeration of nanoparticles continues to occur as the nanoparticles are suspended in the base liquid, the tested particle size is greater than the particle size observed by FE-SEM and TEM (Figure 1 and 2).

**Figure 7:**
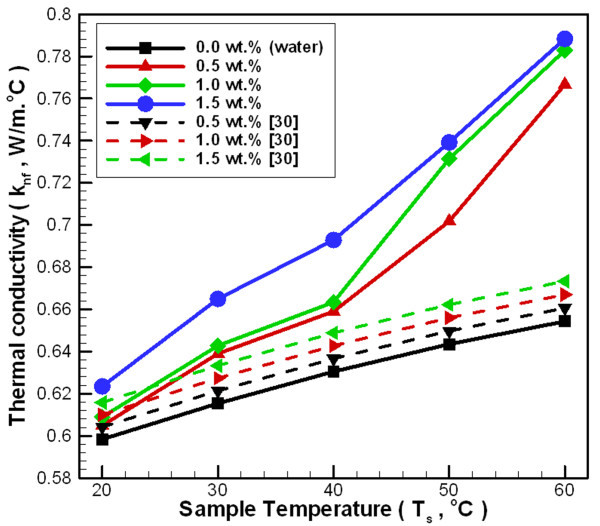
**Thermal conductivity of Al_2_O_3_/water nanofluid at various temperatures and concentrations**.

Figure 7 depicts the changes in thermal conductivity for nanofluid at various temperatures and concentrations over a temperature range of 20°C to 60°C. This figure reveals that as the temperature increases, the effect of increasing nanoparticle concentration on the thermal conductivity ratio is lower than changing the applied temperature. Increasing both the concentration and temperature raises the probability that nanoparticle-liquid collisions will produce a near quasi-convection phenomenon. Increasing random collision behavior helps increase the thermal conductivity of Al_2_O_3_/water nanofluid. However, some researchers believe that these factors do not cause a significant increase in thermal conductivity [33,34]. For a concentration of 0.5 wt.% and a temperature in the range of 20°C to 60°C, the thermal conductivity ratio increases by 1.1% to 17.2%. For concentration of 1.0 wt.%, the thermal conductivity ratio increases by 1.8% to 19.7%. For a concentration of 1.5 wt.%, the thermal conductivity ratio increases by 4.2% to 20.5% compared to water.

Figure 7 also reveals an underestimation between the Pak and Cho's model [30] and the current experimental results. The nanoparticle volume fraction was transformed into the nanoparticle weight fraction using the true density of nanoparticles to unify the concentration of units (Equation 6). Pak and Cho's model [30] was originally obtained with a temperature of 300 K, a particle size of 13 nm, and a concentration range of 1.34-4.33 vol.%. Because this model does not incorporate changes of temperature and particle size, it originally obtained at a higher concentration, and its deviation is a little higher. However, considering the uncertainty of the experiment in this study, this deviation is within an acceptable range under 20°C to 30°C.

**Figure 8:**
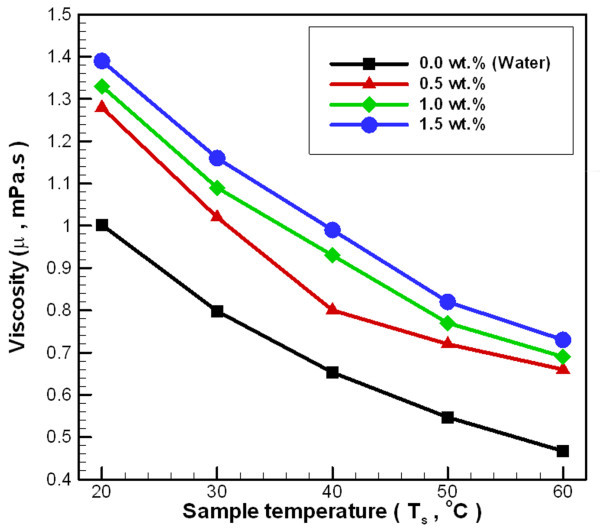
**Viscosity of Al_2_O_3_/water nanofluid at various temperatures and concentrations**.

Figure 8 depicts the changes in viscosity for Al_2_O_3_/water nanofluid at various temperatures and concentrations. In general, the nanofluid viscosity increases with increasing nanoparticle loading in the base liquid. For a concentration of 0.5 wt.% and within a temperature range of 20°C to 60°C, the viscosity ratio increases by 21.5% to 41.3%. For a concentration of 1.0 wt.%, the viscosity ratio increases by 32.7% to 47.8%. For a concentration of 1.5 wt.%, the viscosity ratio increases by 38.7% to 56.3%. These results show that the viscosity of Al_2_O_3_/water nanofluid is much higher than water. The pressure drop of pipeline-related issues must be considered when the Al_2_O_3_/water nanofluid is applied to heat exchange.

**Figure 9:**
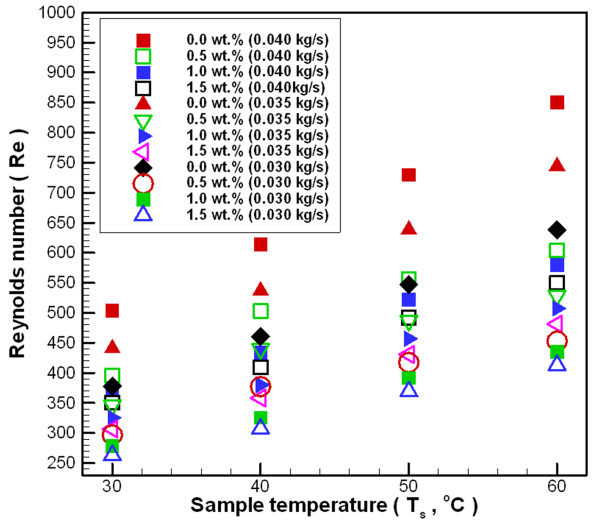
**Reynolds number of Al_2_O_3_/water nanofluid at various temperatures and concentrations under different mass flow rates**.

Figure 9 shows the change in Reynolds number (Re) for Al_2_O_3_/water nanofluid at various temperatures and concentrations for different mass flow rates. This figure reveals that at the same mass flow rate, Re increases with the increasing temperature of nanofluid, but Re decreases with the increasing concentration of nanofluid. The whole experimental range of Re is limited to the laminar flow range (< < 2000). In general, the viscosity (Figure 8) and density (Equation 4) of nanofluid increases with increasing nanoparticle loading in the base liquid, and the viscosity ratio of the nanofluid is greater than the enhanced density ratio of the nanofluid. At the same mass flow rate, the higher density of the fluid leads to a lower flow velocity. Thus, the Re of the nanofluid will be lower than water at the same mass flow rate and temperature conditions.

**Figure 10:**
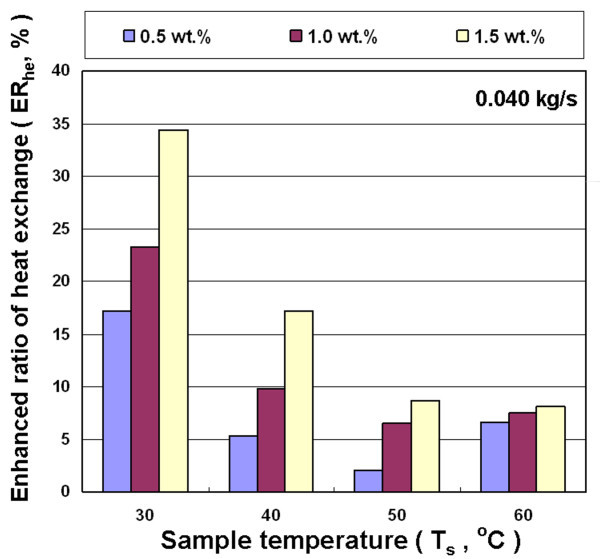
**Enhanced heat exchange ratio for Al_2_O_3_/water nanofluid for different concentrations and temperatures at 0.040 kg/s**.

**Figure 11:**
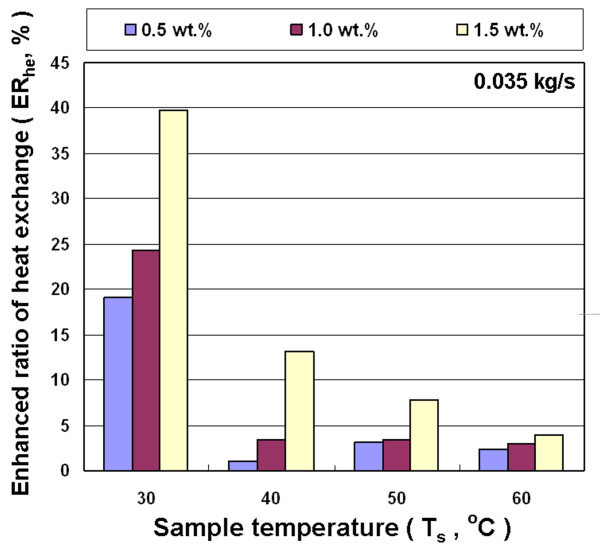
**Enhanced heat exchange ratio for Al_2_O_3_/water nanofluid for different concentrations and temperatures at 0.035 kg/s**.

**Figure 12:**
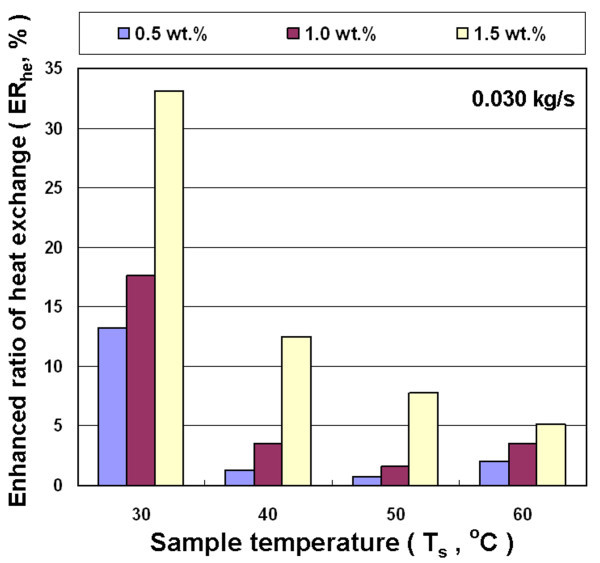
**Enhanced heat exchange ratio for Al_2_O_3_/water nanofluid for different concentrations and temperatures at 0.030 kg/s**.

Figures 10, 11, and 12 show the effects of different concentration, inlet temperature, and mass flow rates of nanofluid on the enhanced ratio of heat exchange capacity (ER_he_). Results show that nanofluid can enhance the air-cooled heat exchange capacity ratio under all experimental conditions investigated in this study. This is primarily because the added nanoparticles improved the heat transfer performance of the fluid. The addition of nanoparticles reveals the following heat exchange enhancement mechanism: (a) Because nanoparticles have higher thermal conductivity, a higher concentration of nanoparticles results in a more obvious heat conduction enhancement. (b) Nanoparticle collisions with the base fluid molecules and the wall of the heat exchanger strengthen energy transmission. (c) The nanofluid increases friction between the fluid and the heat exchanger wall, and thus improves heat exchange capacity. On the above factors that influence the heat exchange capacity, the collision of these nanoparticles strengthens the movement of nanoparticles suspended in fluid due to higher temperature and the increased mass flow rate of fluid. Furthermore, the higher temperature and mass flow rate strengthen the collision of nanoparticles with the wall of heat exchanger. These effects influence the functions of the heat exchanger.

Figures 10, 11, and 12 reveal that ER_he _decreases with increasing temperature of nanofluid at different mass flow rates, but the concentration of nanofluid increases with increased ER_he_. These results show that the enhanced ratio of heat exchange decreases at higher temperature. This seems to contradict the statement above that a high temperature increases the probability of collision between nanoparticles and liquid molecules, which can increase heat exchange. This contradictory phenomenon is mainly because the heat exchanger used in this study is a rectangular tube with a great cross-section aspect ratio (*W*/*H *= 17.3/1.9). The flow rate distribution of the fluid with higher viscosity is relatively uneven in such cross-section. Thus, the effective cross-sectional area of the pipe for heat exchange was decreased to decrease the enhanced ratio of heat exchange. Figure 8 shows that the decrease rate of viscosity of the nanofluid is lower than water at higher temperatures. This means that the viscosity of water is much lower than nanofluid at high temperatures. This strengthens the phenomenon of uneven flow rate distribution in the rectangular tube with a great aspect ratio, which in turn enhances the ratio of heat exchange at higher temperatures lower than the lower temperature for nanofluid. The maximum enhanced ratio of heat exchange was obtained at 30°C and 1.5 wt.% for various mass flow rates, and are about 33-39% compared with water. In addition, under various experimental conditions, a higher concentration of nanofluid led to an enhanced ratio of heat exchange increases.

**Figure 13:**
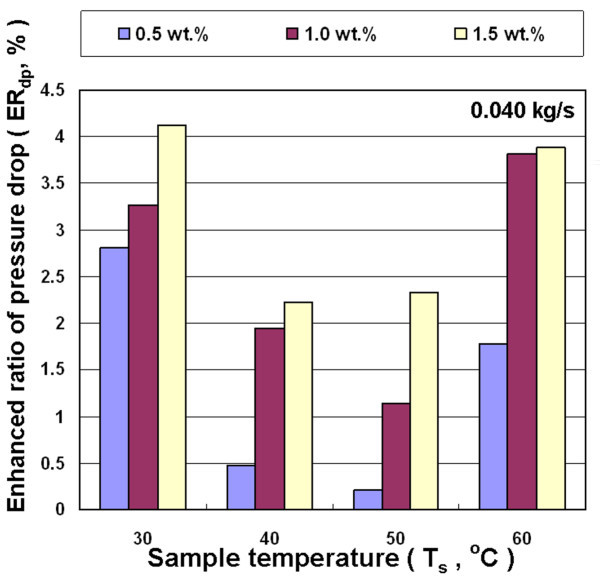
**Enhanced pressure drop ratio for Al_2_O_3_/water nanofluid for different concentrations and temperatures at 0.040 kg/s**.

**Figure 14:**
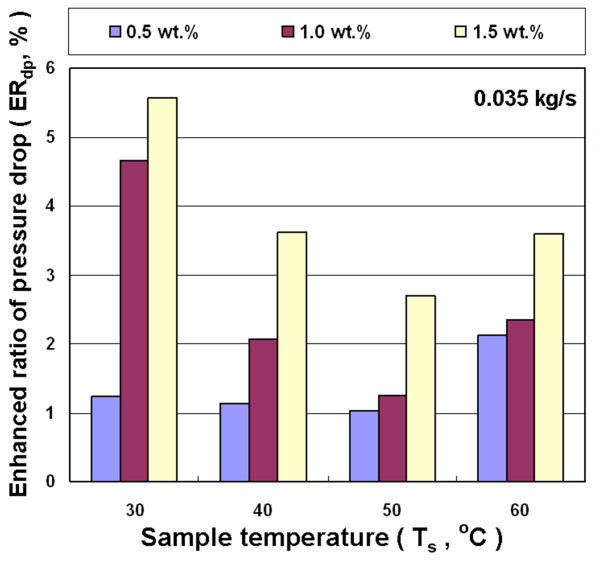
**Enhanced pressure drop ratio for Al_2_O_3_/water nanofluid for different concentrations and temperatures at 0.035 kg/s**.

**Figure 15:**
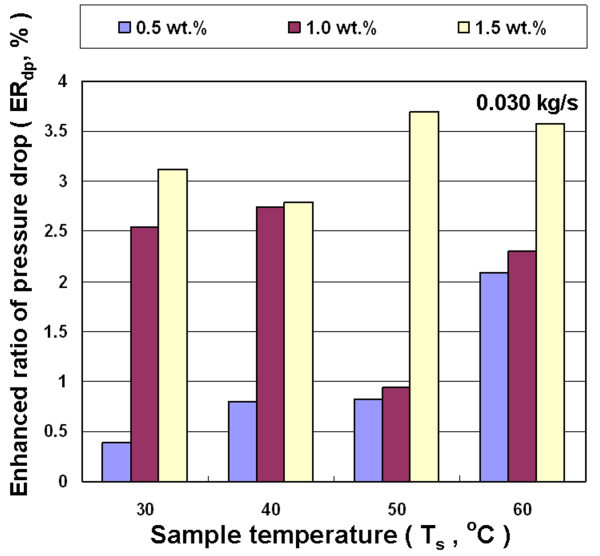
**Enhanced pressure drop ratio for Al_2_O_3_/water nanofluid for different concentrations and temperatures at 0.030 kg/s**.

Figures 13, 14, and 15 show the effects of different nanofluid concentration, inlet temperature, and mass flow rates on the enhanced ratio of pressure drop (ER_dp_). In general, the viscosity of nanofluid increases with increasing nanoparticle loading in the base liquid, and has a higher friction factor. The pressure drop experiment in this study shows a higher concentration of nanofluid for a higher enhanced ratio of pressure drop at different temperatures and mass flow rates. However, there is not a significant trend between the enhanced ratio of pressure drop and either the flow rate or temperature. In the whole range of experimental parameters, the largest enhanced ratio of pressure drop was 5.6%, occurring at the temperature of 30°C, mass flow rate of 0.035 kg/s and the concentration of 1.5 wt.%. The experiments on heat exchange and pressure drop show that the overall benefits significantly decrease when nanofluid is used in air-cooled heat exchanger at higher temperature. The enhanced ratio of pressure drop becomes even higher than the enhanced ratio of heat exchange under some conditions, which leads to an overall efficiency of cooling system using nanofluid that is lower than that using water. This is primarily because the enhanced ratio of heat exchange is lower at higher temperature. Therefore, the air-cooled heat exchanger operating at 30-40°C has the best overall efficiency in this study.

## Conclusions

This study analyzes the characteristics of Al_2_O_3_/water nanofluid to determine the feasibility of its application in an air-cooled heat exchanger under laminar flow. Results confirm that Al_2_O_3_/water nanofluid offers a higher heat exchange capacity than water, and a higher concentration of nanoparticles provides an even greater enhancement ratio of the heat exchange. At higher temperature, however, the nanofluid does not provide greater enhanced ratio of the heat exchange due to rectangular tube with a large cross-section aspect ratio and enhanced viscosity ratio. In the whole range of experimental parameters in this study, the maximum enhanced ratio of heat exchange and pressure drop was approximately 39% and 5.6%, respectively. The air-cooled heat exchanger operating at 30-40°C had the best overall efficiency. Therefore, the temperature and mass flow rate of the working fluid can affect the enhanced ratio of heat exchange and pressure drop of nanofluid in addition to the nanoparticle concentration. The cross-section aspect ratio of the tube in the heat exchanger is also an important factor to be taken into consideration.

## Competing interests

The authors declare that they have no competing interests.

## Authors' contributions

TPT, YHH, and TCT designed the experiment. TPT and YHH fabricated the sample. TPT, YHH, and JHC carried out the measurements. TPT, YHH, TCT, and JHC analyzed the measurements. TPT, YHH, and TCT wrote the paper. All authors read and approved the final manuscript.
